# Among Five First-Trimester Complete Blood Count Inflammatory Indices, the Systemic Inflammation Response Index Rises Most Consistently with the Severity of Preterm Birth

**DOI:** 10.3390/diagnostics16162553

**Published:** 2026-08-13

**Authors:** Aybekcan Batman, Enes Saraç, Ali Konu, Esra Selvi, Yücel Kaya, Murat İbrahim Toplu, Burak Deniz Aydoğdu, Gökhan Bolluk

**Affiliations:** 1 Division of Perinatology, Department of Obstetrics and Gynaecology, Başakşehir Çam and Sakura City Hospital, 34480 Istanbul, Türkiye; konuali@gmail.com (A.K.); esraorak@yahoo.com.tr (E.S.); yucelkaya0007@gmail.com (Y.K.); drgbolluk@hotmail.com (G.B.); 2 Department of Obstetrics and Gynaecology, Başakşehir Çam and Sakura City Hospital, 34480 Istanbul, Türkiye; dr.enessarac@gmail.com; 3 Department of Obstetrics and Gynaecology, Prof. Dr. Cemil Taşçıoğlu City Hospital, 34384 Istanbul, Türkiye; mitoplu@gmail.com (M.İ.T.); burakd.1992@hotmail.com (B.D.A.)

**Keywords:** preterm birth, systemic inflammation response index, systemic immune-inflammation index, inflammatory indices, complete blood count, monocytes

## Abstract

**Background/Objectives:** Complete blood count inflammatory indices are widely studied in obstetrics but rarely compared directly. We compared five first-trimester indices, the systemic immune-inflammation index (SII), systemic inflammation response index (SIRI), pan-immune-inflammation value (PIV), neutrophil-to-lymphocyte ratio (NLR), and platelet-to-lymphocyte ratio (PLR), for their associations with preterm birth, examined across its severity. **Methods:** In a retrospective single-centre cohort of 5003 singleton pregnancies with a first-trimester complete blood count, late (34–36 weeks) and early (<34 weeks) preterm births were analysed separately. The association of each index with preterm birth was assessed by logistic regression, per standard deviation, and by quartile, with adjustments made for maternal age, body mass index, parity, fetal sex, and gestational age at sampling; discrimination was compared using the DeLong test. The birthweight category, classified against the INTERGROWTH-21st standards, was a secondary outcome. **Results:** After adjustment, all indices except for the PLR were associated with preterm delivery, with similar effects per standard deviation. Among the five indices, the SIRI showed the most consistent association with the severity of preterm birth: its values rose at every step from term to late to early birth. It discriminated these births best (area under the curve 0.600, significantly greater than the SII), and its quartile gradient was steepest, with the odds more than doubling from the lowest to the highest quartile. All five remained weak discriminators, with every area under the curve being below 0.70, and none was associated with the birthweight category. **Conclusions:** These indices are not standalone predictive tests, but among them, the SIRI, which, unlike the SII, captures the monocyte count, was most closely associated with early, more severe preterm birth, consistent with the role of monocytes in the inflammation of labour.

## 1. Introduction

Preterm birth, defined as delivery before 37 completed weeks of gestation, is the leading cause of death in children under five years of age. About 13.4 million infants were born preterm worldwide in 2020, roughly one in ten live births [1]. Reported rates vary widely between countries, from roughly 5% to 18% of live births [2]. Infants who survive face higher risks of early neonatal complications such as respiratory distress syndrome, intraventricular haemorrhage, necrotising enterocolitis, and retinopathy of prematurity [3], together with long-term sequelae, including neurodevelopmental impairment and cardiometabolic disease [4]. These risks are not uniform across the preterm range; they rise steeply as gestational age falls, so early preterm infants (<34 weeks) carry substantially higher mortality and severe morbidity than late preterm infants (34–36 weeks) [5,6].

Preterm labour is a final common pathway with several distinct causes [7], and inflammation is one of its central drivers, whether triggered by intra-amniotic infection and chorioamnionitis or by sterile, non-infectious signals [8]. The maternal innate immune system is activated ahead of term, and leukocytes, mainly neutrophils and monocytes/macrophages, infiltrate the decidua, fetal membranes, cervix, and myometrium, where they release pro-inflammatory cytokines such as interleukin-1β, interleukin-6, and tumour necrosis factor-α, together with prostaglandins and reactive oxygen species [4,9]. These mediators promote cervical ripening, activate the fetal membranes, and switch the myometrium to a contractile phenotype, producing coordinated uterine contractions [10]. The response is not confined to the uterus: maternal circulating neutrophils, monocytes, and lymphocytes are activated and shift in their relative proportions during pregnancy and in the period before labour, giving inflammation a measurable systemic signature [11].

Because this systemic inflammatory state can be captured from a routine, inexpensive complete blood count, several derived indices have been proposed: the neutrophil-to-lymphocyte ratio (NLR; neutrophils/lymphocytes), the platelet-to-lymphocyte ratio (PLR; platelets/lymphocytes), the systemic immune-inflammation index (SII; platelets × neutrophils/lymphocytes), the systemic inflammation response index (SIRI; neutrophils × monocytes/lymphocytes), and the pan-immune-inflammation value (PIV; neutrophils × platelets × monocytes/lymphocytes). The three composite indices, SII [12], SIRI [13], and PIV [14], were first defined as cancer prognostic markers. These, together with the simpler ratios, have since been applied to obstetric conditions such as pre-eclampsia, gestational diabetes, fetal growth restriction, and pregnancy loss [15,16,17,18]. They differ in one respect that matters here: the SIRI and PIV include the absolute monocyte count, whereas the NLR, PLR, and SII do not. This distinction is relevant because monocytes are central to the inflammatory pathway of labour: circulating maternal monocytes infiltrate the myometrium and decidua, where they differentiate into macrophages and help propagate the cytokine and chemokine signalling that leads to myometrial contraction [19,20]. An index that weights the monocyte count may therefore reflect this pathway more closely than one that omits it.

Several studies have examined these indices in relation to preterm birth, but the findings are mixed and often conflicting [21]. Much of this work rests on modest or selected samples, such as women already admitted with threatened preterm labour [22] rather than asymptomatic pregnancies, and the studies vary in the timing of blood sampling and in how preterm delivery is defined [23,24]. Direct, within-cohort comparisons of all five indices are scarce, and the monocyte-containing indices SIRI and PIV have been studied less than the NLR, PLR, and SII [25].

We therefore compared the NLR, PLR, SII, SIRI, and PIV, measured in the first trimester, for their association with subsequent preterm delivery. The setting was a large, consecutively sampled single-centre cohort of singleton pregnancies. We examined this association across the severity of preterm birth (early, <34 weeks; late, 34–36 weeks) and tested whether it persisted after adjustment for maternal age, body mass index, and parity. We hypothesised that the monocyte-containing indices SIRI and PIV, and SIRI in particular, would discriminate early, more severe preterm delivery better than the SII.

## 2. Materials and Methods

### 2.1. Study Design and Setting

This was a retrospective cohort study conducted at Başakşehir Çam and Sakura City Hospital, a tertiary referral centre in Istanbul, Türkiye. Hospital records over the period 2020 to 2026 were reviewed, and delivery records were linked to first-trimester complete blood count (CBC) results obtained at the routine combined first-trimester screening visit and stored in the hospital information system.

### 2.2. Ethics

This study was approved by the No. 1 Clinical Research Ethics Committee of Başakşehir Çam and Sakura City Hospital (decision no. 111, 11 March 2026; protocol code 2026-111) and was conducted in accordance with the Declaration of Helsinki. Given the retrospective design and the use of anonymised routine clinical records, the committee waived the requirement for informed consent.

### 2.3. Study Population

Singleton pregnancies with a first-trimester CBC and a recorded singleton birth at the centre were eligible. To ensure that the first-trimester indices reflected baseline inflammation, pregnancies with a chronic condition present at the time of sampling that independently affects systemic inflammation were excluded: pregestational (type 1 or type 2) diabetes, chronic hypertension, autoimmune or chronic inflammatory disease, chronic infection, known malignancy, and haematological disease. To avoid acute confounding of the indices, women with clinical signs of an active infection (for example, an upper respiratory or urinary tract infection) or taking antibiotic or anti-inflammatory medication at the time of the first-trimester CBC were also excluded. Twin and higher-order pregnancies were also excluded. Conditions that arose after first-trimester sampling, such as gestational diabetes and pre-eclampsia, were not used as exclusion criteria. The final cohort comprised 5003 singleton pregnancies. Maternal age, body mass index (BMI), parity, fetal sex, and gestational age at the time of the CBC were retrieved as covariates. The BMI was recorded for 3371 pregnancies. Analyses requiring covariate adjustment were therefore restricted to the complete-case subset (*n* = 3370; one further record lacked another covariate).

### 2.4. Laboratory Measurements and Inflammatory Indices

The first-trimester CBC was performed between 11 and 14 weeks of gestation on an automated haematology analyser as part of routine antenatal care. Absolute neutrophil, lymphocyte, monocyte, and platelet counts (×10^9^/L) were used to derive five indices: the neutrophil-to-lymphocyte ratio (NLR = neutrophils/lymphocytes), the platelet-to-lymphocyte ratio (PLR = platelets/lymphocytes), the systemic immune-inflammation index (SII = platelets × neutrophils/lymphocytes), the systemic inflammation response index (SIRI = neutrophils × monocytes/lymphocytes), and the pan-immune-inflammation value (PIV = neutrophils × platelets × monocytes/lymphocytes).

### 2.5. Outcomes and Definitions

Gestational age was determined from the last menstrual period and confirmed by first-trimester ultrasound (crown–rump length), with the ultrasound estimate used when the two were discordant; for pregnancies conceived by in vitro fertilisation, gestational age was based on the embryo transfer date. The primary outcome was preterm delivery, defined as birth before 37 completed weeks and further classified by severity as early (<34 weeks) or late (34–36 weeks) preterm. The secondary outcome was birthweight category, classified as small (SGA, <10th centile), appropriate (AGA), or large (LGA, >90th centile) for gestational age and sex using the INTERGROWTH-21st Newborn Size standards [26], with the very-preterm size reference [27] applied to births before 33 weeks, implemented in R with the gigs package [28]. The INTERGROWTH-21st standards were chosen because they are prescriptive, describing optimal growth from healthy, well-nourished pregnancies under a common protocol; therefore, classification as small or large for gestational age does not depend on the characteristics of the local population.

### 2.6. Statistical Analysis

Analyses were performed in R version 4.6.0 (R Core Team, 2026). Continuous variables are summarised as medians (interquartile range) and categorical variables as counts (percentages). The CBC-derived indices were right-skewed and non-normally distributed; thus, nonparametric methods were used throughout. The three outcome groups (term, late preterm, or early preterm) were compared using the Kruskal–Wallis test, with Dunn’s test and Bonferroni correction for pairwise comparisons; two-group comparisons used the Mann–Whitney U test. The association of each index with preterm delivery was assessed by logistic regression and expressed as the odds ratio per one-standard-deviation increase in the index, with each index standardised to its standard deviation in the analysis sample and modelled separately. Because the indices are mathematically interrelated and share component cell counts, each was entered in a separate model rather than together to avoid collinearity; for the same reason, the indices were compared with one another through their discrimination, described below, rather than as competing terms in a single model. To allow for a possible non-linear relationship, each index was additionally divided into quartiles and the highest quartile compared with the lowest. Adjusted models additionally included maternal age, BMI, parity, fetal sex, and gestational age at the CBC and were fitted on the complete-case subset (*n* = 3370). Because BMI was missing for about one third of women, two further analyses examined the effect of this missingness: the adjusted models were refitted, omitting the BMI, and were refitted on the full cohort without a BMI adjustment (Supplementary Table S1); women with and without a recorded BMI were also compared (Supplementary Table S3). Missing body mass index values were also multiply imputed by predictive mean matching, with 30 imputations. The adjusted models were refitted on each imputed dataset and the estimates pooled by Rubin’s rules (Supplementary Table S1). Discrimination was assessed by the area under the receiver operating characteristic curve (AUC) with 95% confidence intervals, comparing early preterm births, and separately, late preterm births, with term births (the other preterm stratum being excluded from each comparison); AUCs were compared using the DeLong test [29], as implemented in the pROC package [30]. The association models used all other births as the reference for early preterm birth, whereas the discrimination analyses compared each preterm stratum with term births. The comparison of the SIRI with the SII—the comparison specified by our hypothesis—was the primary discrimination comparison; the remaining pairwise DeLong comparisons were secondary and were interpreted cautiously in view of the multiple comparisons made. The per-index regression models were likewise not adjusted for multiple comparisons and were interpreted as exploratory; their *p*-values are nominal. The overall model fit was summarised by Nagelkerke’s R^2^. For the secondary outcome, the association of each index with birthweight category was assessed in the same way, modelling small-for-gestational-age and large-for-gestational-age births (each versus the remainder) by logistic regression with the same covariate adjustment. A two-sided *p*-value below 0.05 was considered statistically significant.

## 3. Results

### 3.1. Cohort Characteristics

**Figure 1:**
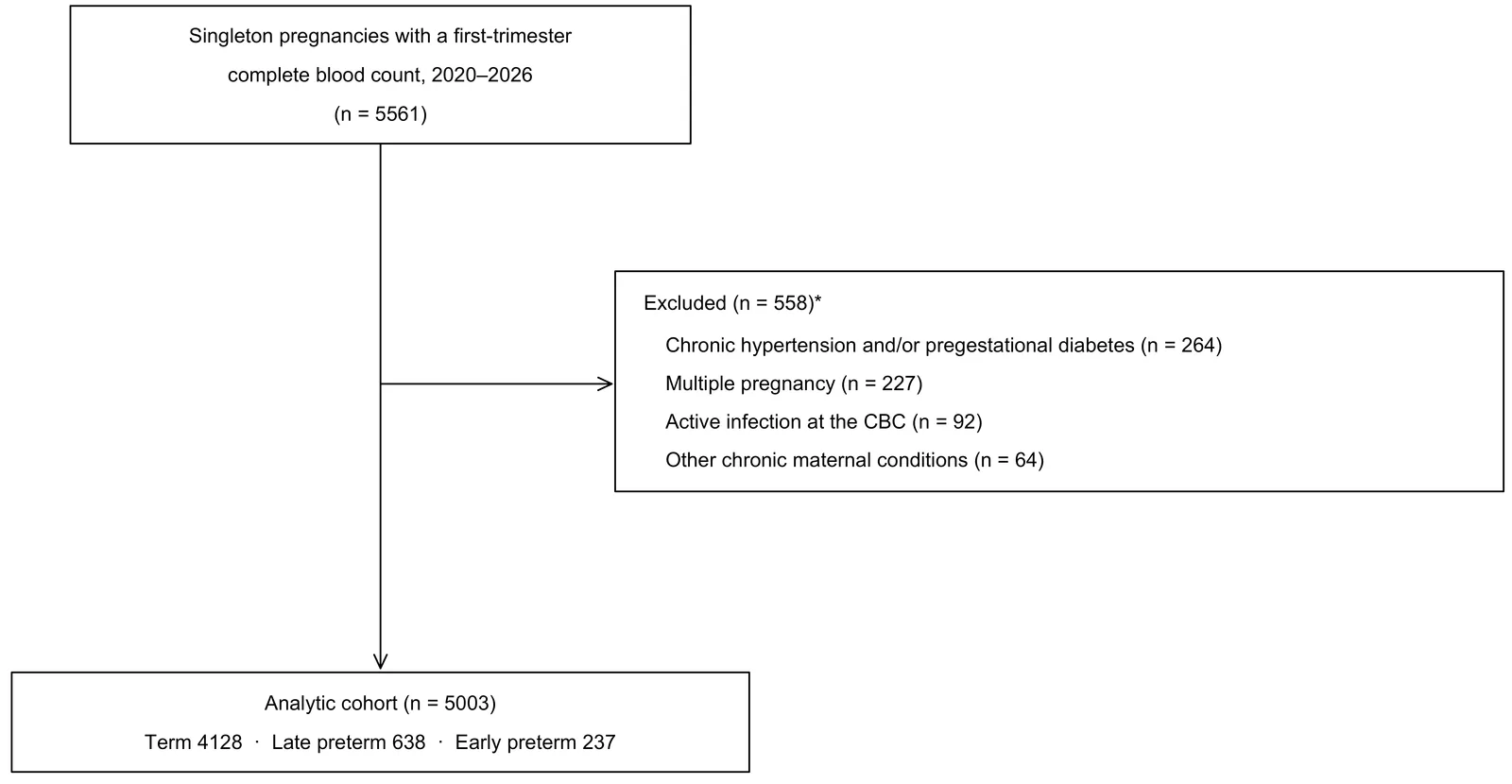
Flow of pregnancies through the study. Selection of the analytic cohort of 5003 singleton pregnancies, showing the exclusions applied and the distribution across term, late preterm (34–36 weeks), and early preterm (<34 weeks) births. * Some pregnancies met more than one exclusion criterion; the counts for the individual categories therefore exceed the total number of pregnancies excluded, each of which was removed only once.

After the exclusion criteria were applied, 5003 singleton pregnancies formed the analytic cohort (Figure 1). Of these, 4128 (82.5%) delivered at term, 638 (12.8%) had a late preterm birth (34–36 weeks), and 237 (4.7%) had an early preterm birth (<34 weeks). Maternal age rose modestly across the three groups, from a median of 28.5 years at term to 29.7 years in the early preterm group (*p* < 0.001) and was retained as a covariate. The body mass index, fetal sex, and gestational age at the first-trimester CBC were similar between groups, whereas parity differed statistically (*p* = 0.014) despite similar medians (Table 1). Birthweight and Apgar scores fell with increasing prematurity, as expected. When newborn size was classified against the INTERGROWTH-21st standards, the proportion of small-for-gestational-age infants increased with the severity of preterm birth (6.1% at term, 10.0% in late preterm, and 12.2% in early preterm births), while large-for-gestational-age infants became less frequent (13.8%, 12.4%, and 10.5%, respectively; *p* < 0.001).

### 3.2. Inflammatory Indices Across Gestational-Age Groups

Median SII, SIRI, PIV and NLR were each higher with greater prematurity (Kruskal–Wallis *p* < 0.001 for all four), whereas PLR was similar across the three groups (*p* = 0.645; Table 2). For SIRI, the median rose from 1.74 (IQR 1.28–2.37) at term to 1.88 (1.37–2.57) in late preterm and 2.06 (1.53–2.81) in early preterm births.

### 3.3. Severity Gradient Across the Preterm Strata

**Figure 2:**
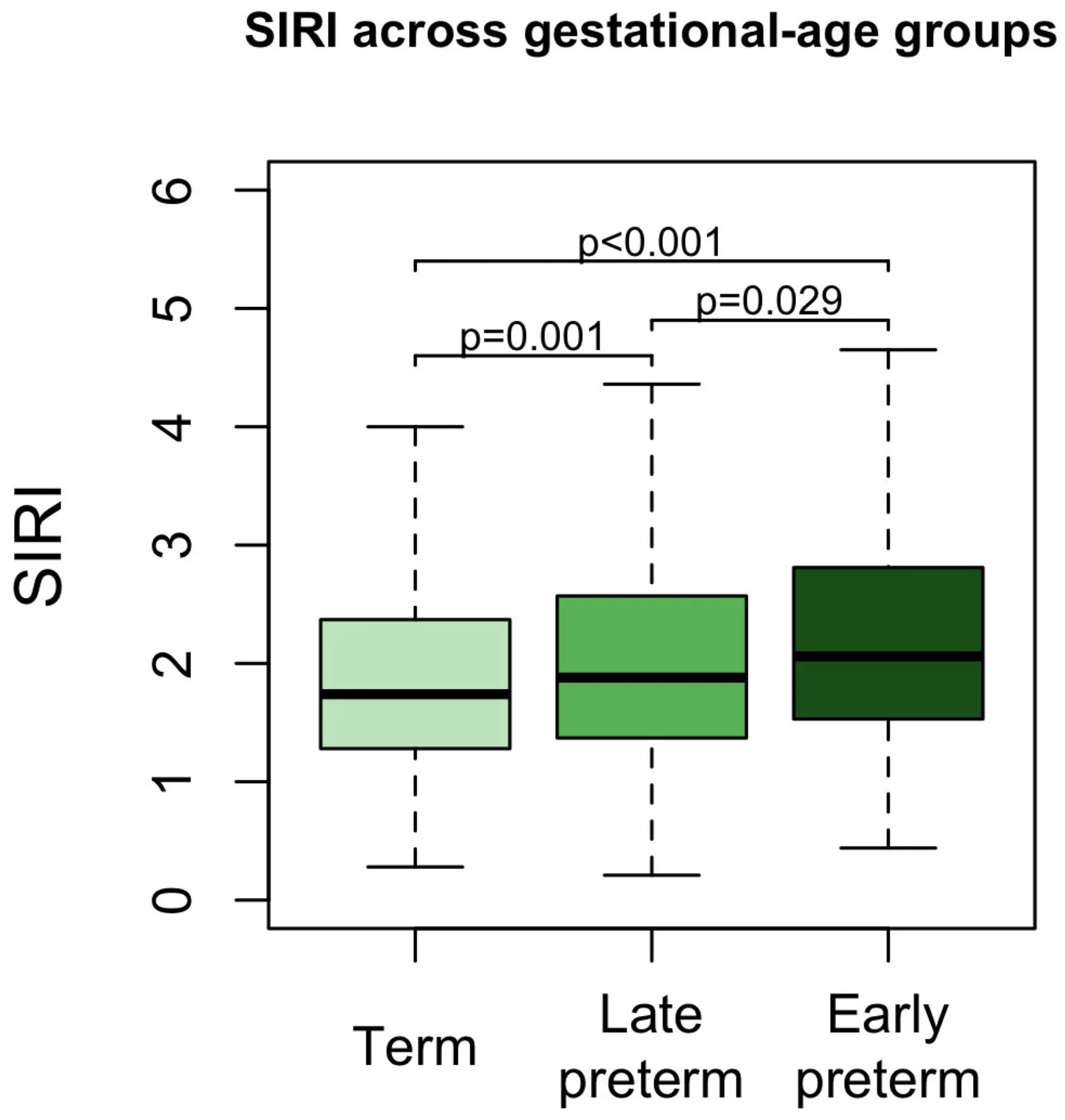
First-trimester systemic inflammation response index (SIRI) across gestational-age groups. Box-and-whisker plots of the SIRI in term (*n* = 4128), late preterm (*n* = 638), and early preterm (*n* = 237) births; the central line shows the median, the box the interquartile range, and the whiskers 1.5 times the interquartile range, with outliers not shown. The graded increase across the three groups illustrates its association with the severity of preterm birth. Pairwise *p*-values are from Dunn’s test with Bonferroni corrections.

Although four indices increased across the groups, only the SIRI separated the two preterm strata from one another. On pairwise comparison, the SIRI differed between term and late preterm (*p* = 0.001), between term and early preterm (*p* < 0.001), and between late and early preterm births (*p* = 0.029), so that each step along the severity gradient was accompanied by a further rise in the index (Figure 2). For the SII, PIV, and NLR, the difference between late and early preterm did not reach significance (*p* = 0.95, 0.24, and 0.22, respectively), and the PLR did not differ at any step. The SIRI was therefore the only index to track the severity of preterm birth in a graded manner.

### 3.4. Adjusted Association with Preterm Birth

Models were adjusted for maternal age, body mass index, parity, fetal sex, and gestational age at the CBC. A one-standard-deviation increase in the SII, SIRI, PIV, and NLR was associated with higher odds of a preterm birth, with closely similar effect sizes (adjusted odds ratio of 1.17 to 1.20; *p* < 0.001 for each). The PLR was not associated with preterm birth (adjusted odds ratio of 1.06, 95% CI 0.97–1.16). The same four indices were associated with preterm birth on univariate testing (Table 3, Panel A). For early preterm births, the SII, SIRI, and PIV remained associated after adjustment (SII *p* = 0.031, SIRI *p* = 0.012, PIV *p* = 0.007), the NLR was of borderline significance (1.14, 95% CI 0.996–1.30; *p* = 0.057), and the PLR showed no association. The adjusted odds ratios did not distinguish the four positive indices from one another, and the SIRI did not have the largest effect size (Supplementary Figure S1); the Nagelkerke R^2^ of each model was low throughout (around 0.02 for the any-preterm models and up to about 0.03 for early preterm birth), which is consistent with a weak overall explanatory value. These adjusted associations were not sensitive to the missing body mass index data. Omitting the body mass index left the per-standard-deviation odds ratios essentially unchanged. Refitting the models on the full cohort without body mass index adjustment reproduced the same pattern, with, if anything, stronger statistical significance (early preterm birth: SIRI 1.19, PIV 1.18, SII 1.15, NLR 1.14; all *p* ≤ 0.008; Supplementary Table S1). Multiple imputation of the missing body mass index gave the same result. The pooled odds ratios per standard deviation for early preterm births were 1.18 (95% CI 1.08–1.30) for the SIRI, 1.18 (1.08–1.30) for the PIV, 1.14 (1.03–1.26) for the SII, and 1.13 (1.02–1.25) for the NLR; the PLR remained unassociated. Confidence intervals were narrower than in the complete-case analysis, reflecting the larger sample, and on this basis, the association of the NLR reached conventional significance (*p* = 0.017).

Comparing the highest with the lowest quartile of each index made the pattern clearer for early preterm birth (Table 3, Panel B). The absolute rate of early preterm birth rose across the SIRI quartiles from 2.9% in the lowest to 7.2% in the highest, and women in the highest SIRI quartile had more than twice the adjusted odds of early preterm birth compared with the lowest (2.31, 95% CI 1.37–3.88). The corresponding contrast for the SII was weaker and did not reach significance (1.42, 95% CI 0.88–2.29); the PIV and NLR were intermediate (1.67 and 1.86), and the PLR showed no gradient. The four positive indices were all associated with any preterm birth on this quartile contrast (adjusted odds ratio 1.50 to 1.86). The quartile gradient was also unaffected by the missing body mass index. After multiple imputation, the contrast for early preterm birth remained steepest for the SIRI (2.57, 95% CI 1.73–3.82), ahead of the PIV (2.11, 1.41–3.16), NLR (1.84, 1.24–2.72), and SII (1.57, 1.07–2.30). The contrast for the SII did not reach significance in the complete-case analysis but did so after imputation, while remaining well below that of the SIRI. The PLR again showed no gradient.

### 3.5. Discrimination

**Figure 3:**
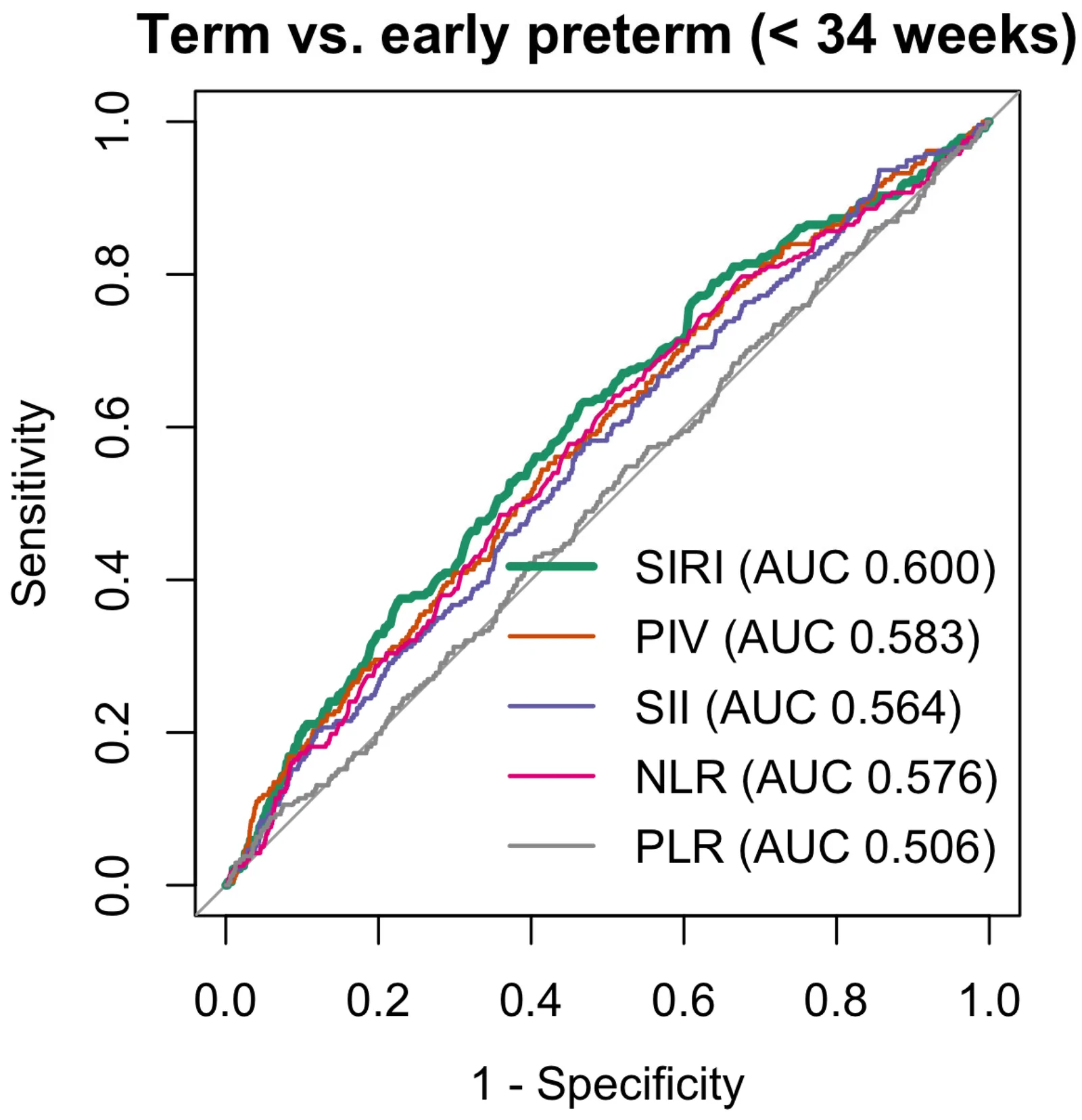
Receiver operating characteristic curves for early preterm birth. Curves for the five first-trimester inflammatory indices (SII, SIRI, PIV, NLR, and PLR) discriminating early preterm birth (<34 weeks) from term birth; late preterm births were excluded from this comparison. Areas under the curve and pairwise comparisons using DeLong’s test are reported in Table 4.

Discrimination depended on the severity of preterm birth, with each preterm stratum being compared with term births. For early preterm birth, the SIRI had the highest area under the curve (0.600, 95% CI 0.562–0.637). In our primary, hypothesis-driven comparison, the SIRI discriminated significantly better than the SII (0.564; DeLong *p* = 0.010). The remaining comparisons were secondary. The SIRI’s area under the curve also exceeded that of the PIV (0.583), NLR (0.576), and PLR (0.506). Once the multiple comparisons were taken into account, however, only the difference from the PLR remained significant (*p* < 0.001), whereas the unadjusted differences from the PIV (*p* = 0.049) and NLR (*p* = 0.069) did not (Table 4; Figure 3). For late preterm births, none of the indices discriminated meaningfully (with an area under the curve of 0.51 to 0.54), and the SIRI held no advantage over the SII (*p* = 0.89). In every comparison, the area under the curve remained below 0.70.

### 3.6. Birthweight Category (Secondary Outcome)

For the secondary outcome, none of the indices was associated with the birthweight category. After adjustment, the odds of small-for-gestational-age birth did not change with any index (adjusted odds ratio 0.90 to 0.96, all *p* ≥ 0.16), and neither did the odds of large-for-gestational-age birth (1.04 to 1.09, all *p* > 0.05); the areas under the curve were close to 0.50 throughout (Supplementary Table S2). The indices were therefore associated with the timing of birth but not with fetal size.

## 4. Discussion

In this large cohort of singleton pregnancies with a first-trimester complete blood count, four of the five indices were associated with preterm delivery, the exception being PLR. The monocyte-containing SIRI tracked early, more severe preterm births most consistently, in three respects. It was the only index whose values rose at every step of the severity gradient, including from late to early preterm birth. Its area under the curve for early preterm birth was the highest, and significantly greater than that of the SII in our primary comparison; it showed the steepest gradient across quartiles: the odds of early preterm birth more than doubled from the lowest quartile to the highest. These signals were specific to early preterm birth; for late preterm births, no index discriminated meaningfully and the SIRI held no advantage.

The advantage of the SIRI should be described carefully. After adjustment, the per-standard-deviation odds ratios of the four positive indices were closely similar, and the SIRI did not have the largest effect; on this scale, the SII was marginally higher. The SIRI’s advantage therefore lies not in the magnitude of its association but in its pattern: it tracked the severity of preterm births in a graded manner, separated early from late preterm births, and discriminated early preterm births more sharply than the SII. The quartile analysis showed this more clearly than the per-standard-deviation models. This fits a relationship that is concentrated at the high end of the distribution rather than spread evenly across it. Read together, the analyses point to a difference in how the SIRI relates to the severity of preterm birth, not to a uniformly stronger marker.

A monocyte-related mechanism offers a coherent explanation. The two indices that distinguished early preterm birth—the SIRI and PIV—both weight the absolute monocyte count, whereas the SII and PLR, which did not track severity, do not; the NLR was intermediate. Maternal monocytes are central to the inflammatory pathway of labour. They are recruited to the myometrium, decidua, cervix, and fetal membranes, where they differentiate into macrophages; there, they release cytokines, chemokines, and matrix metalloproteinases that drive cervical remodelling and myometrial contraction [9,10,20]. Monocytes appear to be the principal cellular drivers of this process: in co-culture, they elicit a large, synergistic inflammatory response from myometrial cells, while neutrophils have only a limited effect [19], and in experimental models, macrophages, but not neutrophils, are required for parturition [10]. The circulating immune cells that these indices sample also show pro-inflammatory changes ahead of preterm births [11]. Among women with threatened preterm labour, the monocyte count and the monocyte-containing indices were highest in those who delivered soonest; composite indices have been argued to predict imminent delivery more reliably than the monocyte count alone [22]. Maternal monocytes are progressively activated in the circulation as pregnancy advances [31], and a systemic rise in circulating monocytes marks the onset of labour [32]. The peripheral monocyte signal that a complete blood count captures therefore may reflect the local recruitment described above. An index that weights the circulating monocyte count may therefore reflect this pathway more closely than one that omits it, which is consistent with the severity gradient here being strongest for the monocyte-containing indices. The PIV also weights the monocyte count, yet it did not separate the preterm strata as sharply as the SIRI. The difference may lie in what else each index contains. The PIV multiplies the monocyte count by the platelet count as well as the neutrophil count, and platelets vary for many reasons unrelated to inflammation. The SIRI weights monocytes against lymphocytes alone, so the monocyte signal may come through more directly. The confidence intervals of the two indices overlap, so their ranking should not be over-interpreted.

The indices were associated with the timing of birth but not with the birthweight category. If these indices simply marked generally high-risk or placentally compromised pregnancies, we would expect an association with small-for-gestational-age birth, since fetal growth restriction is itself largely placental in origin. Instead, the indices were selective for the timing of birth and not for fetal size. This fits a signal that arises from the maternal inflammatory pathway driving labour rather than from impaired placentation. Conditions such as pre-eclampsia and gestational diabetes develop after first-trimester sampling and share inflammatory mechanisms; rather than confounders, they may lie on the causal pathway between the indices and preterm birth and were therefore not treated as exclusions or covariates. We could not, however, formally assess such mediation. This should be read cautiously, as the indices were weak discriminators throughout, and the number of growth-restricted infants was modest; thus, the contrast is suggestive rather than definitive.

The clinical implications are modest and should not be overstated. Although the associations were consistent, discrimination was weak throughout, with every area under the curve below 0.70, and each index explained little of the variation in risk (Nagelkerke R^2^ 0.02 to 0.03). A higher SIRI marks a higher average risk, with the rate of early preterm birth rising from 2.9% in the lowest quartile to 7.2% in the highest, yet it cannot identify which individual pregnancy will deliver early. These indices are therefore best understood as markers of an underlying inflammatory process and as possible components of a multivariable model, not as standalone screening tests. On its own, none of them reach the discrimination required for clinical screening or individual risk stratification.

Our findings sit within a sizeable but inconsistent body of literature, and the disagreement becomes easier to interpret once the studies are separated by when blood was sampled. Two types of study dominate. The first measures the indices acutely in women already admitted with threatened preterm labour and relates them to delivery within days. The second, like ours, measures them in unselected pregnancies long before any symptoms and relates them to preterm birth weeks or months later. These designs ask different questions: one focuses on the inflammation associated with established labour, while the other examines a predisposition that exists well before it. A single large cohort comparing all five indices directly therefore helps to clarify these conflicting reports.

Among the studies that sample acutely, the results are strikingly divergent. In spontaneous preterm labour sampled at its onset, neither the SIRI nor SII separated the groups [21], which fits our interpretation that a first-trimester signal reflects an early predisposition rather than the acute inflammation of established labour. Where the indices did discriminate, the monocyte-containing and the neutrophil-driven ones changed places from one study to the next. In one cohort of women with threatened preterm labour, the monocyte count and the monocyte-containing indices (the SIRI, PIV, and lymphocyte-to-monocyte ratio) were raised in those who delivered soonest, whereas the SII, NLR, and PLR were not [22]. In another, prospectively studied cohort, the opposite held: the NLR and SII were the only independent predictors, and the SIRI, PIV, and the lymphocyte-to-monocyte ratio dropped out after adjustment [5]. Two similar studies reaching opposite conclusions show how unstable the ranking of these indices is in small, selected samples. Near-perfect discrimination has occasionally been reported in this setting [3]. Such reports share the same limitations: a small number of already-labouring women and an outcome defined as delivery within a week. They are therefore best read in that light rather than as a benchmark that our first-trimester values should match.

The studies that, like ours, sample in the first trimester give a more consistent picture, and it is the one our data support. The largest, in more than 70,000 low-risk pregnancies, found very weak discrimination for the NLR, PLR, and the lymphocyte-to-monocyte ratio and did not examine the composite indices [33]. The cohort closest to ours in design was retrospective and single-centre, with blood sampled in the first trimester and preterm birth stratified by severity. It reported that the SIRI was not associated with preterm births overall, yet it rose steeply across the severity strata and was highest in the earliest births [34]. The agreement is partial rather than complete. That cohort found no overall association, whereas we did (adjusted odds ratio 1.19 for any preterm birth). Our larger cohort may explain the difference. Our study extends this first-trimester evidence with a large, unselected cohort, adds the four-component PIV, and provides the first formal, head-to-head comparison of all five indices. The strongest support for our central observation is that two independent first-trimester cohorts, ours and Kırat’s, both locate the SIRI signal in early, severe preterm birth. We are therefore careful not to claim that the SIRI is a generally superior marker. Across the literature, the most informative index varies. Our result is specific: among the five indices measured in the first trimester, the SIRI tracks the severity of preterm birth most closely and discriminates the earliest births most sharply.

A single first-trimester complete blood count is also a labile measure. The cell counts behind these indices shift with transient, largely subclinical influences: minor intercurrent infection, physiological stress, hydration, and ordinary diurnal and biological variations. Any single result is a snapshot rather than a stable maternal characteristic; relating it to a delivery that follows months later is biologically demanding. This temporal variability is a genuine limitation and is one reason why discrimination was modest. It does not, however, make the signal spurious. Across more than five thousand pregnancies, the index rose at each step, from term to late to early preterm birth. Such an ordered gradient is unlikely to arise by chance from a single noisy measurement, since random fluctuation would not produce a graded association of this kind. Because we sampled in the first trimester, long before any clinical signs, these indices capture a distal signal. A modest association measured at such a distance is the expected and honest result, not a failure of the marker.

This study has strengths and limitations. Its strengths are a large, consecutively sampled and unselected cohort, sampling in the first trimester before symptoms, the preservation of the true event rate, classification of newborn size against the INTERGROWTH-21st standards, and a formal head-to-head comparison of discrimination. However, there are several limitations. The cohort came from a single tertiary referral centre, which may over-represent higher-risk pregnancies and limit generalisability. The median body mass index of the cohort was 29.5 kg/m^2^, which is higher than in many published obstetric populations. Since obesity is itself a chronic inflammatory state, the findings should be extrapolated with caution to populations of a lower average body mass index. Data on smoking, a relevant confounder, were not available. Smoking is associated with both preterm birth and systemic inflammation, so this is a genuine source of residual confounding, and we cannot judge the direction of any resulting bias. The relationship may differ in pregnancy: in a cohort of more than 70,000 pregnancies that did record smoking, smokers had lower rather than higher NLRs and PLRs—the reverse of the association seen outside pregnancy [33]. That single observation is not enough to dismiss the confounding, and cohorts with recorded smoking status are needed. We adjusted for measured maternal factors but cannot exclude residual or unmeasured confounding. Because we used the full cohort with covariate adjustment rather than matching, the analysis controls only for the confounders that were measured; this choice preserved the true event rate and statistical power. Early preterm birth, although our primary focus, was the smallest stratum (*n* = 237), so the early-preterm and quartile estimates are less precise than those for any preterm birth, with correspondingly wider confidence intervals. The body mass index, one of the adjustment variables, was missing for about a third of the cohort. Pregnancies with and without a recorded body mass index were compared directly (Supplementary Table S3). Missingness was not at random: the body mass index was recorded less often in pregnancies that ended preterm, so the complete-case analysis under-represented preterm births. Reassuringly, the per-standard-deviation associations were essentially unchanged when the body mass index was omitted and when the models were refitted on the full cohort, indicating that the findings were not an artefact of this missingness.

We could not distinguish spontaneous from medically indicated preterm births, as the dataset did not record the onset of labour, membrane status, or indication for delivery; the association we describe is therefore with all-cause early preterm birth. This matters for interpretation because the monocyte-related mechanism we propose applies to the spontaneous, inflammation-driven pathway rather than to deliveries performed for maternal or fetal indications. The signal may therefore be driven largely by spontaneous births, though this remains inferential. Most preterm births are spontaneous: spontaneous labour and preterm prelabour rupture of membranes together account for about two-thirds of cases, and these are regarded as an inflammation- and infection-related syndrome [7]. Raised indices in this group would be expected and consistent with our interpretation. The conditions that most often prompt indicated preterm delivery, chiefly pre-eclampsia and fetal growth restriction, typically co-occur with small-for-gestational-age birth, yet no index was associated with small-for-gestational-age birth in our cohort, which argues against a signal dominated by such deliveries. We cannot, however, exclude some contribution from indicated births, and confirmation in cohorts that separate the preterm phenotypes is needed. Finally, the retrospective design carries the usual risks of incomplete and non-standardised data.

## 5. Conclusions

In conclusion, complete blood count inflammatory indices have become popular in obstetric research because they are inexpensive, routinely available, and simple to calculate, and a growing body of literature has proposed them as markers of a preterm birth. Our results temper that enthusiasm. In a large first-trimester cohort, none of the five indices discriminated preterm birth well enough to serve as a clinical decision-making tool on its own, and each explained only a small fraction of the variation in risk. What the data do support is narrower. The monocyte-containing indices, and the SIRI in particular, were associated with early, more severe preterm births more specifically than the SII. The risk also rose appreciably across the SIRI distribution: the highest quartile carried more than twice the odds of an early preterm birth of the lowest. Read this way, these indices are best understood not as standalone predictive tests but as inexpensive markers of an inflammatory contribution to early preterm birth. Their extreme values may add information within a broader, multivariable assessment. Of the five indices, the SIRI tracked early preterm births most consistently, a finding that is consistent with a monocyte-related inflammatory signal, which warrants confirmation in prospective, multicentre cohorts.

## Figures and Tables

**Table 1:** Maternal and neonatal characteristics by gestational-age group. Values are median (interquartile range) or *n* (%). Body mass index was recorded for 3371 pregnancies. Birthweight was classified against the INTERGROWTH-21st Newborn Size standards as small (SGA), appropriate (AGA) or large (LGA) for gestational age and sex. Continuous variables were compared with the Kruskal–Wallis test and categorical variables with the χ^2^ test. CBC, complete blood count.

Characteristic	Term (*n* = 4128)	Late Preterm (*n* = 638)	Early Preterm (*n* = 237)	*p*
Maternal age, years	28.5 (25.1–32.8)	29.6 (25.5–34.2)	29.7 (26.0–35.2)	<0.001
Body mass index, kg/m^2^	29.5 (26.8–32.9)	29.7 (26.2–34.2)	29.4 (26.2–32.5)	0.467
Parity	2 (1–3)	2 (1–3)	2 (1–3)	0.014
Gestational age at CBC, days	87 (81–93)	87.5 (82–93)	87 (81–93)	0.334
Male sex, *n* (%)	2122 (51.4)	312 (48.9)	131 (55.3)	0.225
Birthweight, g	3270 (3000–3560)	2630 (2311–2900)	1510 (1100–1970)	<0.001
Apgar score, 1 min	8 (7–8)	8 (7–8)	6 (4–7)	<0.001
Apgar score, 5 min	9 (9–9)	9 (8–9)	8 (6–8)	<0.001
Birthweight category				<0.001
SGA, *n* (%)	252 (6.1)	64 (10.0)	29 (12.2)	
AGA, *n* (%)	3306 (80.1)	495 (77.6)	183 (77.2)	
LGA, *n* (%)	570 (13.8)	79 (12.4)	25 (10.5)	

**Table 2:** First-trimester inflammatory indices by gestational-age group. Values are median (interquartile range). The *p*-values are from the Kruskal–Wallis test across the three groups; pairwise Dunn–Bonferroni comparisons are reported in the text and shown in Figure 2. SII, systemic immune-inflammation index; SIRI, systemic inflammation response index; PIV, pan-immune-inflammation value; NLR, neutrophil-to-lymphocyte ratio; PLR, platelet-to-lymphocyte ratio.

Index	Term (*n* = 4128)	Late Preterm (*n* = 638)	Early Preterm (*n* = 237)	*p*
SII	791 (609–1051)	839 (633–1101)	864 (665–1139)	<0.001
SIRI	1.74 (1.28–2.37)	1.88 (1.37–2.57)	2.06 (1.53–2.81)	<0.001
PIV	435 (300–623)	474 (322–700)	506 (352–747)	<0.001
NLR	3.18 (2.55–4.02)	3.32 (2.62–4.19)	3.51 (2.84–4.38)	<0.001
PLR	131 (109–160)	133 (111–162)	132 (110–161)	0.645

**Table 3:** Association of each index with preterm birth. Values are odds ratios (95% confidence interval), with each index modelled separately. Any preterm denotes late and early preterm combined versus term; early preterm denotes birth before 34 weeks versus all other births. In Panel B, each index was divided into quartiles and the highest quartile compared with the lowest. Adjusted models included maternal age, body mass index, parity, fetal sex, and gestational age at the CBC, and were fitted on the complete-case subset (*n* = 3370; one of the 3371 pregnancies with a recorded body mass index lacked a further covariate). Nagelkerke R^2^ for the adjusted per-standard-deviation any-preterm models ranged from 0.016 (PLR) to 0.024 (SII). Abbreviations as in Table 2.

Panel A. Odds Ratio per One-Standard-Deviation Increase in the Index				
**Index**	**Any Preterm, Univariate**	**Any Preterm, Adjusted**	**Early Preterm, Univariate**	**Early Preterm, Adjusted**
SII	1.14 (1.07–1.22)	1.20 (1.10–1.30)	1.13 (1.02–1.25)	1.16 (1.01–1.32)
SIRI	1.16 (1.09–1.24)	1.19 (1.09–1.29)	1.17 (1.07–1.28)	1.18 (1.04–1.35)
PIV	1.16 (1.09–1.24)	1.19 (1.09–1.29)	1.17 (1.06–1.29)	1.20 (1.05–1.36)
NLR	1.13 (1.06–1.20)	1.17 (1.08–1.27)	1.12 (1.02–1.24)	1.14 (0.996–1.30)
PLR	1.05 (0.98–1.12)	1.06 (0.97–1.16)	1.04 (0.92–1.17)	0.95 (0.78–1.15)
**Panel B. Adjusted Odds Ratio for the Highest Versus the Lowest Quartile (Q4 vs. Q1)**				
**Index**	**Any Preterm, Adjusted**		**Early Preterm, Adjusted**	
SII	1.50 (1.15–1.97)		1.42 (0.88–2.29)	
SIRI	1.86 (1.42–2.45)		2.31 (1.37–3.88)	
PIV	1.64 (1.24–2.16)		1.67 (1.02–2.72)	
NLR	1.57 (1.19–2.08)		1.86 (1.09–3.16)	
PLR	1.21 (0.92–1.60)		0.90 (0.54–1.48)	

**Table 4:** Discrimination of each index for preterm birth (area under the receiver operating characteristic curve). Each preterm stratum was compared with term births, with the other stratum excluded. For early preterm birth, DeLong tests against the SIRI gave *p* = 0.010 (SII), 0.049 (PIV), 0.069 (NLR), and <0.001 (PLR); SIRI–SII was the primary hypothesis-driven comparison, while among the secondary comparisons, only SIRI–PLR remained significant once multiple testing was taken into account. For late preterm birth, no index differed significantly from the SIRI (all *p* > 0.48) except for the PLR (*p* = 0.036). AUC, area under the receiver operating characteristic curve. Other abbreviations as in Table 2.

Index	Early Preterm vs. Term, AUC (95% CI)	Late Preterm vs. Term, AUC (95% CI)
SIRI	0.600 (0.562–0.637)	0.543 (0.518–0.567)
PIV	0.583 (0.545–0.620)	0.544 (0.520–0.569)
NLR	0.576 (0.539–0.614)	0.537 (0.513–0.561)
SII	0.564 (0.526–0.601)	0.542 (0.518–0.566)
PLR	0.506 (0.468–0.545)	0.511 (0.487–0.535)

## Data Availability

Because the data derive from individual patient records, they are not held in a public repository for reasons of patient privacy. The anonymised dataset analysed during the current study is available from the corresponding author on reasonable request, subject to institutional and ethics committee approval.

## Supplementary Materials

The following supporting information can be downloaded at: https://www.mdpi.com/article/10.3390/diagnostics16162553/s1. Figure S1: Adjusted odds ratio per one-standard-deviation increase in each index for any preterm birth and for early preterm birth (forest plot); Table S1: Sensitivity of the early-preterm associations to body mass index adjustment, to complete-case restriction, and to multiple imputation; Table S2: Association of each first-trimester index with birthweight category; Table S3: Comparison of pregnancies with and without a recorded first-trimester body mass index.
