# Supplementary material for: Among Five First-Trimester Complete Blood Count Inflammatory Indices, the Systemic Inflammation Response Index Rises Most Consistently with the Severity of Preterm Birth
Source: Diagnostics (Basel). 2026 Aug 13;16(16):2553. doi: 10.3390/diagnostics16162553 (PMC13511698; doi:10.3390/diagnostics16162553)
Supplement: Supplementary file 1 [file diagnostics-16-02553-s001.zip › diagnostics-4432787-supplementary.pdf]

Among the five first-trimester complete blood count inflammatory indices, the systemic inflammation response index rises most consistently with the severity of preterm birth

Aybekcan Batman[1*], Enes Saraç[2], Ali Konu[1], Esra Selvi[1], Yücel Kaya[1], Murat İbrahim Toplu[3], Burak Deniz Aydoğdu[3], Gökhan Bolluk[1]

Supplementary information

Supplementary Figure S1. Adjusted association of each index with preterm birth (forest plot). Adjusted odds ratio per one-standard-deviation increase in each index for any preterm birth and for early preterm birth, with 95% confidence intervals, from the models in Table 3, Panel A.

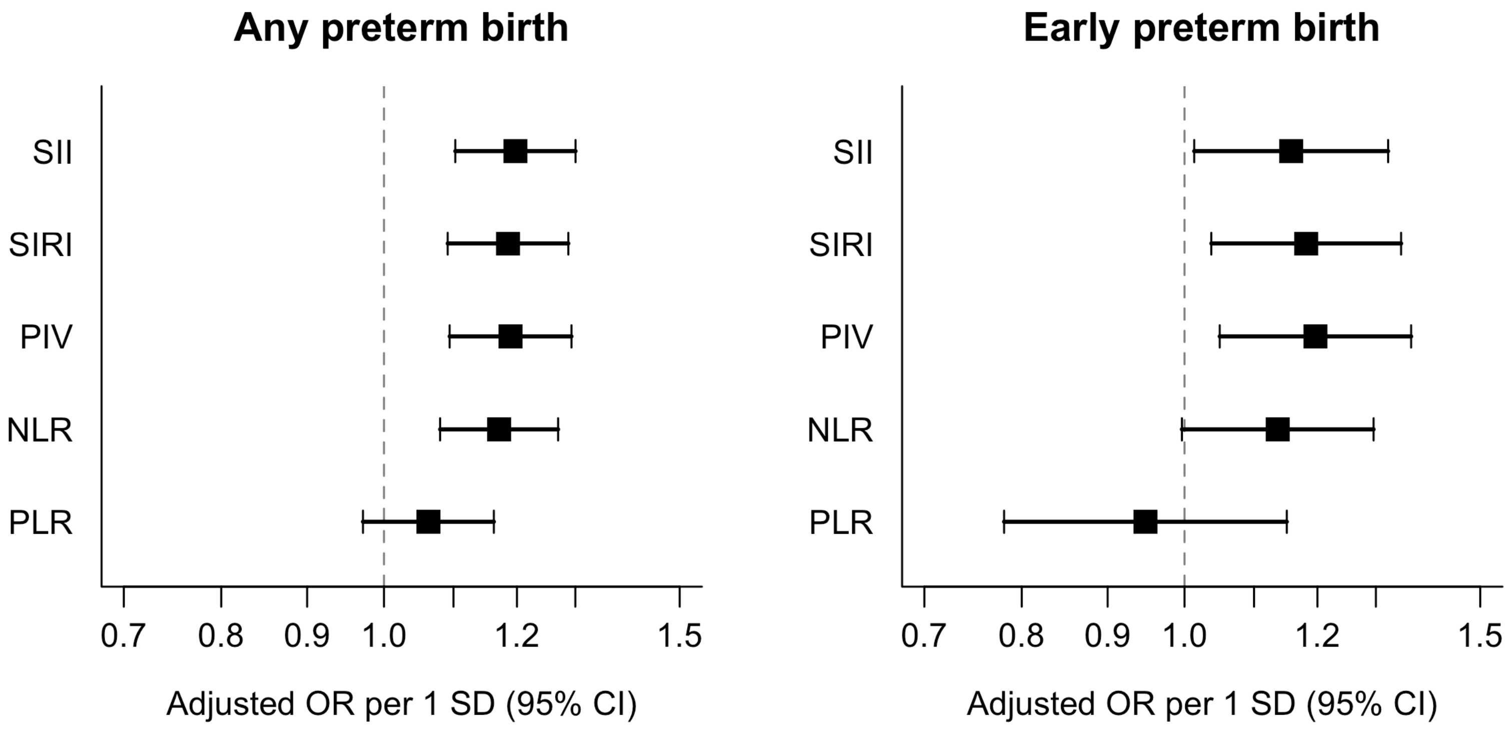

Supplementary Table S1: Sensitivity of the early-preterm associations to body mass index adjustment, to complete-case restriction and to multiple imputation.

Index	Complete-case, BMI-adjusted (n = 3370)	Complete-case, BMI omitted (n = 3370)	Full cohort, BMI omitted (n = 5002)	Multiple imputation, BMI imputed (n = 5002)
SII	1.16 (1.01–1.32), p = 0.031	1.17 (1.02–1.34), p = 0.023	1.15 (1.04–1.27), p = 0.008	1.14 (1.03–1.26), p = 0.011
SIRI	1.18 (1.04–1.35), p = 0.012	1.19 (1.04–1.36), p = 0.009	1.19 (1.08–1.31), p < 0.001	1.18 (1.08–1.30), p < 0.001
PIV	1.20 (1.05–1.36), p = 0.007	1.20 (1.05–1.37), p = 0.007	1.18 (1.08–1.30), p = 0.001	1.18 (1.08–1.30), p < 0.001
NLR	1.14 (0.996–1.30), p = 0.057	1.15 (1.01–1.31), p = 0.032	1.14 (1.04–1.26), p = 0.007	1.13 (1.02–1.25), p = 0.017
PLR	0.95 (0.78–1.15), p = 0.588	0.97 (0.80–1.17), p = 0.757	1.05 (0.93–1.18), p = 0.477	1.03 (0.91–1.16), p = 0.700

Values are adjusted odds ratios (95% confidence interval) for early preterm birth (<34 weeks versus all other births) per one standard deviation of the index, with each index modelled separately and expressed per one standard deviation within its own analysis sample. The first column is the complete-case, body mass index-adjusted model reported in Table 3 (Panel A); the second omits the body mass index from the same complete-case sample; the third refits the model on the full cohort (all pregnancies with the four non-body mass index covariates available) without body mass index adjustment; the fourth reports estimates after multiple imputation of the missing body mass index by predictive mean matching with 30 imputations, pooled by Rubin's rules. Covariates were maternal age, parity, fetal sex, and gestational age at the CBC, with the body mass index added in the first and fourth columns. The per-standard-deviation associations are essentially unchanged across the four models. Confidence intervals in the third and fourth columns are narrower than in the complete-case models because the analysis sample is larger; on this basis, the association of the NLR with early preterm birth, of borderline significance in the complete-case model, reached conventional significance. The full-cohort and imputed models include 5002 of the 5003 pregnancies; one pregnancy lacked a further covariate. Abbreviations as in Table 2.

Supplementary Table S2. Association of each first-trimester index with birthweight category, expressed as the odds ratio per one-standard-deviation increase.

Index	SGA, adjusted OR (95% CI)	SGA p	LGA, adjusted OR (95% CI)	LGA p
SII	0.92 (0.79–1.06)	0.24	1.09 (0.98–1.20)	0.10
SIRI	0.93 (0.81–1.07)	0.33	1.08 (0.98–1.20)	0.12
PIV	0.96 (0.84–1.10)	0.55	1.08 (0.98–1.20)	0.11
NLR	0.90 (0.77–1.05)	0.16	1.08 (0.98–1.19)	0.12
PLR	0.96 (0.84–1.10)	0.56	1.04 (0.94–1.16)	0.42

Values are odds ratios (95% confidence interval) per one-standard-deviation increase in the index, with each index modelled separately and adjusted for maternal age, body mass index, parity, fetal sex, and gestational age at the CBC (complete-case subset, n = 3370). Small (SGA) and large (LGA) for gestational age and sex were classified with the INTERGROWTH-21st Newborn Size standards, each modelled versus the remainder of the cohort. No index was associated with either category (all p > 0.05; exact values shown in the table), and all areas under the curve were between 0.49 and 0.53. Abbreviations as in Table 2.

Supplementary Table S3. Comparison of pregnancies with and without a recorded first-trimester body mass index.

Variable	BMI recorded (n = 3371)	BMI missing (n = 1632)	p*
Maternal age (years)	28.3 (24.8–32.6)	29.3 (25.8–33.8)	<0.001
Gestational age at CBC (days)	87 (81–93)	87 (81–93)	0.36
Parity	2 (1–3)	2 (1–3)	<0.001
SII	798 (613–1071)	808 (616–1046)	0.92
SIRI	1.8 (1.3–2.5)	1.7 (1.3–2.3)	0.084
PIV	445 (308–648)	438 (300–624)	0.17
NLR	3.2 (2.6–4.0)	3.2 (2.6–4.1)	0.96
PLR	131 (109–160)	132 (110–161)	0.41
Male sex	51.3%	51.1%	0.89
Any preterm birth	14.4%	23.8%	<0.001
Early preterm birth	3.8%	6.6%	<0.001

*Continuous variables (median [interquartile range]) were compared with the Mann–Whitney U test and categorical variables (%) with the χ^2 test. The body mass index was recorded for 3371 of 5003 women. Missingness was related to the outcome, in that preterm and early preterm births were more frequent among women without a recorded body mass index, but it was not related to any of the five inflammatory indices (all $p \geq 0.08$). Sensitivity analyses that omitted the body mass index, that refitted the models on the full cohort, and that multiply imputed missing values left the per-standard-deviation associations essentially unchanged (Methods; Results; Supplementary Table S1). Abbreviations as in Table 2.
